# Supplementary material for: Methanotrophy under extreme alkalinity in a serpentinizing system
Source: Nat Commun. 2026 Jun 29;17:8135. doi: 10.1038/s41467-026-72513-6 (PMC13458360; doi:10.1038/s41467-026-72513-6)
Supplement: Supplementary file 1 [file 41467_2026_72513_MOESM12_ESM.pdf]

nature portfolio

Corresponding author(s): Alta E. G. Howells

Last updated by author(s): 01/19/2026

Reporting Summary

Nature Portfolio wishes to improve the reproducibility of the work that we publish. This form provides structure for consistency and transparency in reporting. For further information on Nature Portfolio policies, see our Editorial Policies and the Editorial Policy Checklist.

Statistics

For all statistical analyses, confirm that the following items are present in the figure legend, table legend, main text, or Methods section.

n/a	Confirmed	
☐	☒	The exact sample size (*n*) for each experimental group/condition, given as a discrete number and unit of measurement
☐	☒	A statement on whether measurements were taken from distinct samples or whether the same sample was measured repeatedly
☐	☒	The statistical test(s) used AND whether they are one- or two-sided<br>*Only common tests should be described solely by name; describe more complex techniques in the Methods section.*
☒	☐	A description of all covariates tested
☐	☒	A description of any assumptions or corrections, such as tests of normality and adjustment for multiple comparisons
☐	☒	A full description of the statistical parameters including central tendency (e.g. means) or other basic estimates (e.g. regression coefficient) AND variation (e.g. standard deviation) or associated estimates of uncertainty (e.g. confidence intervals)
☐	☒	For null hypothesis testing, the test statistic (e.g. F, t, r) with confidence intervals, effect sizes, degrees of freedom and P value noted<br>*Give P values as exact values whenever suitable.*
☒	☐	For Bayesian analysis, information on the choice of priors and Markov chain Monte Carlo settings
☒	☐	For hierarchical and complex designs, identification of the appropriate level for tests and full reporting of outcomes
☒	☐	Estimates of effect sizes (e.g. Cohen's *d*, Pearson's *r*), indicating how they were calculated

Our web collection on statistics for biologists contains articles on many of the points above.

Software and code

Policy information about availability of computer code

Data collection	No software or code required for data collection for this study.
Data analysis	Programs and software used for 16S rRNA gene amplicon sequence analysis<br>DADA2 – Used for sequence denoising and resolving amplicon sequence variants (ASVs) at 100% sequence similarity (Callahan et al., 2016).<br>Version not specified.<br>QIIME2 – Used for downstream sequence processing and diversity analyses.<br>Version not specified.<br>q2-diversity (QIIME2 plugin) – Used for alpha diversity analysis.<br>Version not specified.<br>q2-feature-classifier (QIIME2 plugin) – Used for taxonomic assignment with the naïve Bayes classifier.<br>Version not specified.<br>Greengenes 13_8 (99%) 16S rRNA gene database – Used as the reference database for taxonomic classification (DeSantis et al., 2006).<br><br>Programs and software used for shotgun metagenomic sequence analysis<br>DOE Systems Biology Knowledgebase (KBase) – Platform used for data upload, processing, and workflow execution.<br>Version not specified.<br>FastQC – Used for quality assessment of raw and trimmed sequencing reads.<br>Version: v0.12.1<br>Trimmomatic – Used for trimming raw sequencing reads based on quality scores.

Version: v0.36
metaSPAdes – Used for metagenomic assembly of quality-checked reads.
Version: v3.15.3
RASTtk – Used for functional annotation of assembled contigs and selected genome bins using default KBase parameters.
Version: v1.073
CONCOCT – Used for binning assembled contigs into metagenome-assembled genomes (MAGs).
Version: v1.1
MetaBAT2 – Used for binning assembled contigs into MAGs.
Version: v1.7
DAS Tool – Used to optimize and dereplicate bins generated by CONCOCT and MetaBAT2.
Version: v1.1.2
CheckM – Used to assess completeness and contamination of genome bins.
Version: v1.0.18
Extract Bins as Assemblies from BinnedContigs (KBase app) – Used to extract genome bins as individual assemblies.
Version: v1.0.2
GTDB-Tk – Used for taxonomic classification of genome bins.
Version: v1.7.0
Merge Reads Libraries (KBase app) – Used to merge quality-checked reads for co-assembly workflows.
Version: v1.2.2
Bowtie2 – Used to map raw reads to genome assemblies to determine relative bin abundance across sites.
Version: v2.3.2
BLASTp – Used to compare predicted protein sequences against reference methanogen genomes and determine percent identity.
Version not specified.

For manuscripts utilizing custom algorithms or software that are central to the research but not yet described in published literature, software must be made available to editors and reviewers. We strongly encourage code deposition in a community repository (e.g. GitHub). See the Nature Portfolio guidelines for submitting code & software for further information.

Data

Policy information about availability of data

All manuscripts must include a data availability statement. This statement should provide the following information, where applicable:

- Accession codes, unique identifiers, or web links for publicly available datasets
- A description of any restrictions on data availability
- For clinical datasets or third party data, please ensure that the statement adheres to our policy

For raw shotgun metagenome sequencing data please see NCBI SRA BioProject: PRJNA1207711

Research involving human participants, their data, or biological material

Policy information about studies with human participants or human data. See also policy information about sex, gender (identity/presentation), and sexual orientation and race, ethnicity and racism.

Reporting on sex and gender	NA
Reporting on race, ethnicity, or other socially relevant groupings	NA
Population characteristics	NA
Recruitment	NA
Ethics oversight	NA

Note that full information on the approval of the study protocol must also be provided in the manuscript.

Field-specific reporting

Please select the one below that is the best fit for your research. If you are not sure, read the appropriate sections before making your selection.

☐ Life sciences ☐ Behavioural & social sciences ☒ Ecological, evolutionary & environmental sciences

For a reference copy of the document with all sections, see nature.com/documents/nr-reporting-summary-flat.pdf

Ecological, evolutionary & environmental sciences study design

All studies must disclose on these points even when the disclosure is negative.

Study description	The sampling sites in this study are surface expressions or seeps of serpentinized fluid that have undergone varying degrees of mixing with surrounding surface water and extent of equilibration with the atmosphere. The percent serpentinized fluid was calculated using the concentration of Si measured in sampling site fluids and a mixing model as described in Howells et al., 2022. Fluids composed of > 99 % serpentinized fluid are characterized as pristine serpentinized fluid/Type II. The fluid with the highest concentration of Si represents fluid influenced from the surface (surface water/Type I), and sites that have between 0-99% serpentinized fluid are considered mixtures of Type I and Type II fluids. In total, 28 sites from 6 locations (Dima, Qafifah, Shumayt, Falaij North, Falaij South, and Al Bana) are in this study (see Howells et al. (2022) for a map of sampling locations). The aqueous chemistry and dissolved gas concentrations were previously reported in Leong et al. (2021) and Howells et al. (2022). For this study we analyzed δ13C DIC of fluids at all sites. At 17 sites we analyzed δ13CH4. Biological analyses (shotgun metagenome sequencing and 16S rRNA gene amplicon sequencing) were conducted on collections of small pebbles to fine silt sediments collected in surface expression pools as described in Howells et al., (2022). Shotgun metagenome sequencing was performed on DNA extracts from sediments of 3 sites. For assessment of methanotroph community structure at each of our study sites we analyzed 16S rRNA gene sequences published in Howells et al. (2022). For assessment of geochemical parameters that may influence methanotrophs we used geochemical data previously reported in Howells et al. (2022) and Howells et al. (2023). The study sites in Howells et al. (2022) and (2023) are the same sites, sampled at the same time as this study.
Research sample	This study focuses on the microbial ecology and activity of aerobic methanotrophs in serpentinized fluids of the Samail ophiolite, where fluids expressed at the surface can be enriched in CH_4 and interact with the atmosphere, creating natural chemical gradients. Field sampling did not target specific methanotrophic taxa. Instead, to investigate methanotroph communities and the geochemical context in which they occur, we characterized the microbiomes of silt and rock sediments underlying surface-expressed serpentinized fluids. To constrain fluid geochemistry and bulk CH_4 isotope composition, we also sampled the associated serpentinized fluids.
Sampling strategy	Sampling strategy was based on previous gecheochemical and energy availability calculations for methanotrophy reported in Canovas et al. (2017) that showed a gradient in CH4 and electron acceptor concentrations with varying extents of atmospheric influence and fluid mixing. We aimed to collect and analyze fluids and their underlying sediments that would both support methanotrophy and sites that we anticipated would not based on electron acceptor and donor concentration.
Data collection	Field data were recorded in a field notebook and subsequently transferred to a Shock Lab spreadsheet (.xlsx).<br>Ion chromatography data were collected in spreadsheet format, processed, and consolidated into a Shock Lab master spreadsheet (.xlsx). Processed data were then exported as a .csv file formatted to meet the input requirements of the AqEquil modeling program (https://doi.org/10.5281/zenodo.13530217).<br>Gas chromatography data were recorded in a laboratory notebook, transferred to an .xlsx file for processing, and incorporated into the Shock Lab master spreadsheet. These data were subsequently exported as a .csv file for use with the AqEquil modeling program (https://doi.org/10.5281/zenodo.13530217).<br>16S rRNA gene amplicon sequencing data were processed and compiled on a shared Shock Lab server and backed up to an external hard drive. Raw sequencing data were deposited in the NCBI Sequence Read Archive (SRA) under BioProject PRJNA1207711.
Timing and spatial scale	Region: Samail ophiolite in Oman<br>Locations:<br>Dima: latitude (degree): 22.98331; longitude (degree): 58.59418; Days sampled, 01/10/14 and 01/12/14<br>Qafifah: latitude (degree): 22.90472; longitude (degree): 58.42427; Days sampled, 01/11/14<br>Falej South: latitude (degree): 22.83787; longitude (degree): 58.05677; Days sampled, 01/13/14<br>Falej North: latitude (degree): 22.84262; longitude (degree): 58.05805; Days sampled, 01/14/14<br>Al Bana: latitude (degree): 23.29318; longitude (degree): 56.87859; Days sampled, 01/15/14<br>Shumayt: latitude (degree): 23.40602; longitude (degree): 56.86361; Days sampled, 01/16/14 and 01/17/14
Data exclusions	No data was excluded in this study. We note that some gas samples were not analyzed for delta13CH4 due to quantified equilibration with the atmosphere, which would impact isotopic composition of CH4. This is detailed in the methods section for bulk isotopic analysis of CH4.
Reproducibility	Experiments were not conducted in this study.
Randomization	This study did not include experimental subjects and therefore no randomization was applied.
Blinding	This study did not include experimental subjects and therefore blinding was not required.

Did the study involve field work? ☒ Yes ☐ No

Field work, collection and transport

Field conditions	The field trip took place during the winter months in the Samail Ophiolite of Oman, January 01/10/14 to 01/17/2014. There was no rainfall. No cloud cover. Temperature of the seeps varied as noted in the manuscript.

Location	Region: Samail ophiolite in Oman<br>Locations:<br>Dima: latitude (degree): 22.98331; longitude (degree): 58.59418; Days sampled, 01/10/14 and 01/12/14<br>Qafifah: latitude (degree): 22.90472; longitude (degree): 58.42427; Days sampled, 01/11/14<br>Falej South: latitude (degree): 22.83787; longitude (degree): 58.05677; Days sampled, 01/13/14<br>Falej North: latitude (degree): 22.84262; longitude (degree): 58.05805; Days sampled, 01/14/14<br>Al Bana: latitude (degree): 23.29318; longitude (degree): 56.87859; Days sampled, 01/15/14<br>Shumayt: latitude (degree): 23.40602; longitude (degree): 56.86361; Days sampled, 01/16/14 and 01/17/14
Access & import/export	Permissions for seep sampling and export of samples was provided by the The Ministry of Agriculture, Fisheries and Water Resource in the Sultanate of Oman.
Disturbance	Minimal disturbance was imparted on the seep. Approximately 50 grams of sediments (rock and silt) underlying seep fluids were collected in less than a 10 cm squared sampling area at each sampling site.

Reporting for specific materials, systems and methods

We require information from authors about some types of materials, experimental systems and methods used in many studies. Here, indicate whether each material, system or method listed is relevant to your study. If you are not sure if a list item applies to your research, read the appropriate section before selecting a response.

Materials & experimental systems

n/a	Involved in the study
x	Antibodies
x	Eukaryotic cell lines
x	Palaeontology and archaeology
x	Animals and other organisms
x	Clinical data
x	Dual use research of concern
x	Plants

Methods

n/a	Involved in the study
x	ChIP-seq
x	Flow cytometry
x	MRI-based neuroimaging

Plants

Seed stocks	NA
Novel plant genotypes	NA
Authentication	NA
