# Supplementary material for: Methanotrophy under extreme alkalinity in a serpentinizing system
Source: Nat Commun. 2026 Jun 29;17:8135. doi: 10.1038/s41467-026-72513-6 (PMC13458360; doi:10.1038/s41467-026-72513-6)
Supplement: Supplementary file 2 [file 41467_2026_72513_MOESM1_ESM.pdf]

Supplementary Table Captions.

Supplementary Data 1. Concentrations of reactants and products for methane oxidation coupled to O2, NO3- and SO3-2. Estimated energy availability in unite kJ per mole electron and kJ per kg fluid.

Supplementary Data 2. Core methanotroph metabolism genes, annotations and BLASTp results.

Supplementary Data 3. Descriptive statistics and Mann-Whitney U test parameters to assess the significance of distribution differences of aerobic methanotroph phylotypes with respect to total ammonia nitrogen (TAN).

Supplementary Data 4. ASVs classified as methanotrophs, the taxonomic assignment and BLASTn results.

Supplementary Data 5. Relative abundance of ASVs classified as methanotrophs.

Supplementary Data 6. Relative abundance of methanotroph phylotypes by group.

Supplementary Data 7. Bin39 metabolic pathway overview from DRAM annotation.

Supplementary Code 1. .faa file for coding sequences of Bin39 and their DRAM annotations.

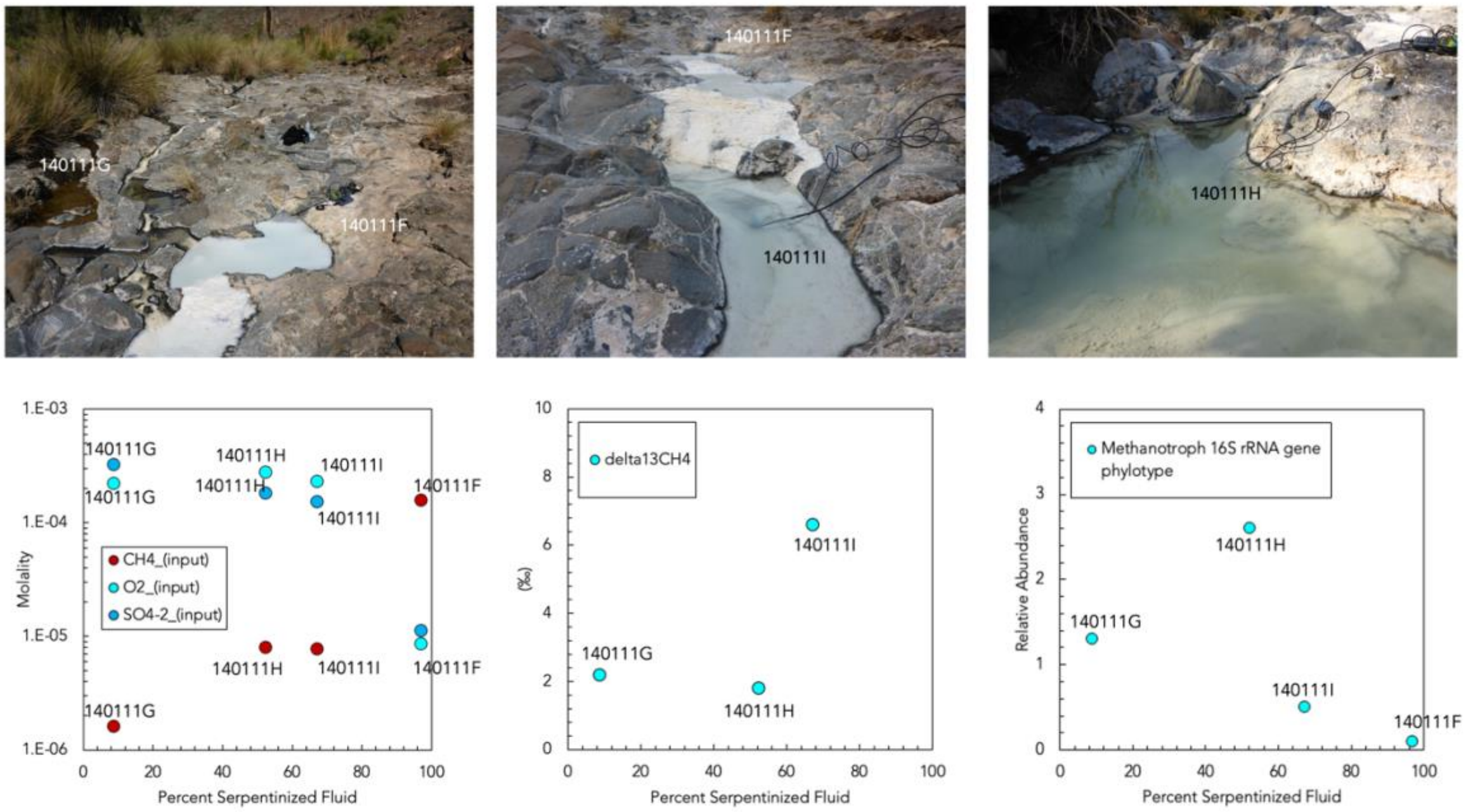

Supplementary Figure 1. Overview of the field location, Qafifah. The sampling locations and corresponding geochemistry, methane isotopes and biology are shown by the sampling site IDs: 140111G, 140111H, 140111I and 140111F.

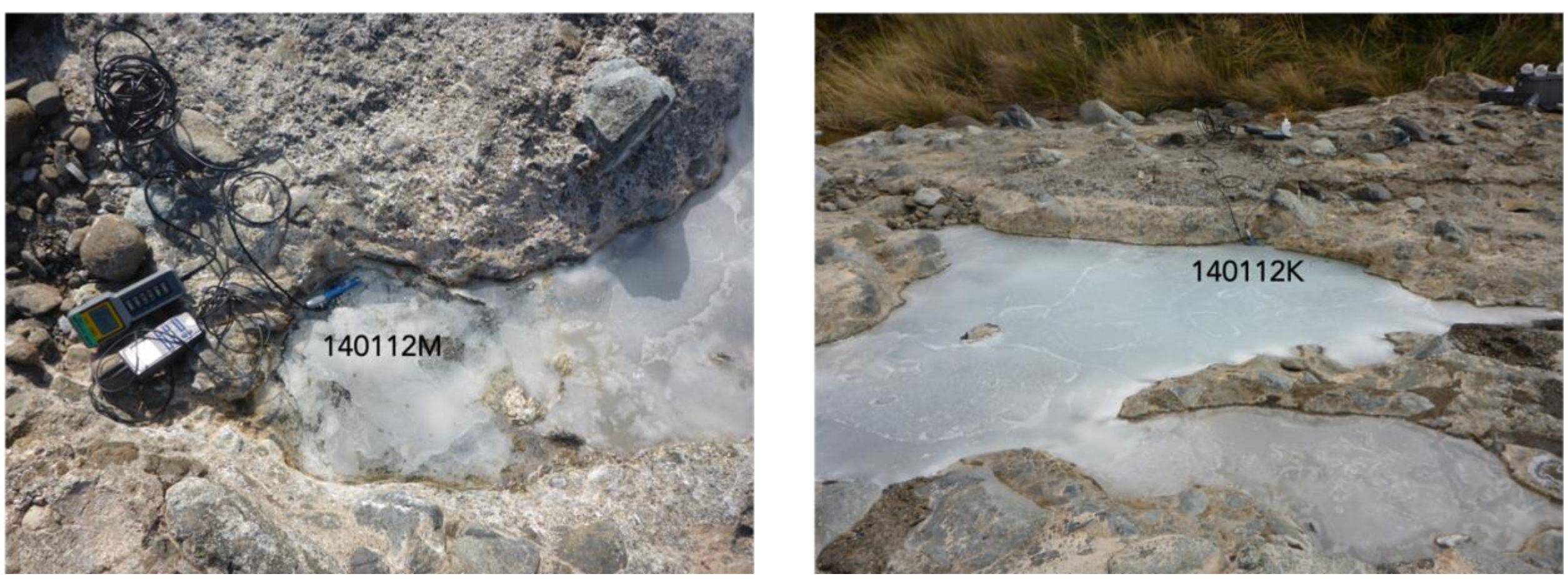

Supplementary Figure 2. Images of sampling sites at Dima.

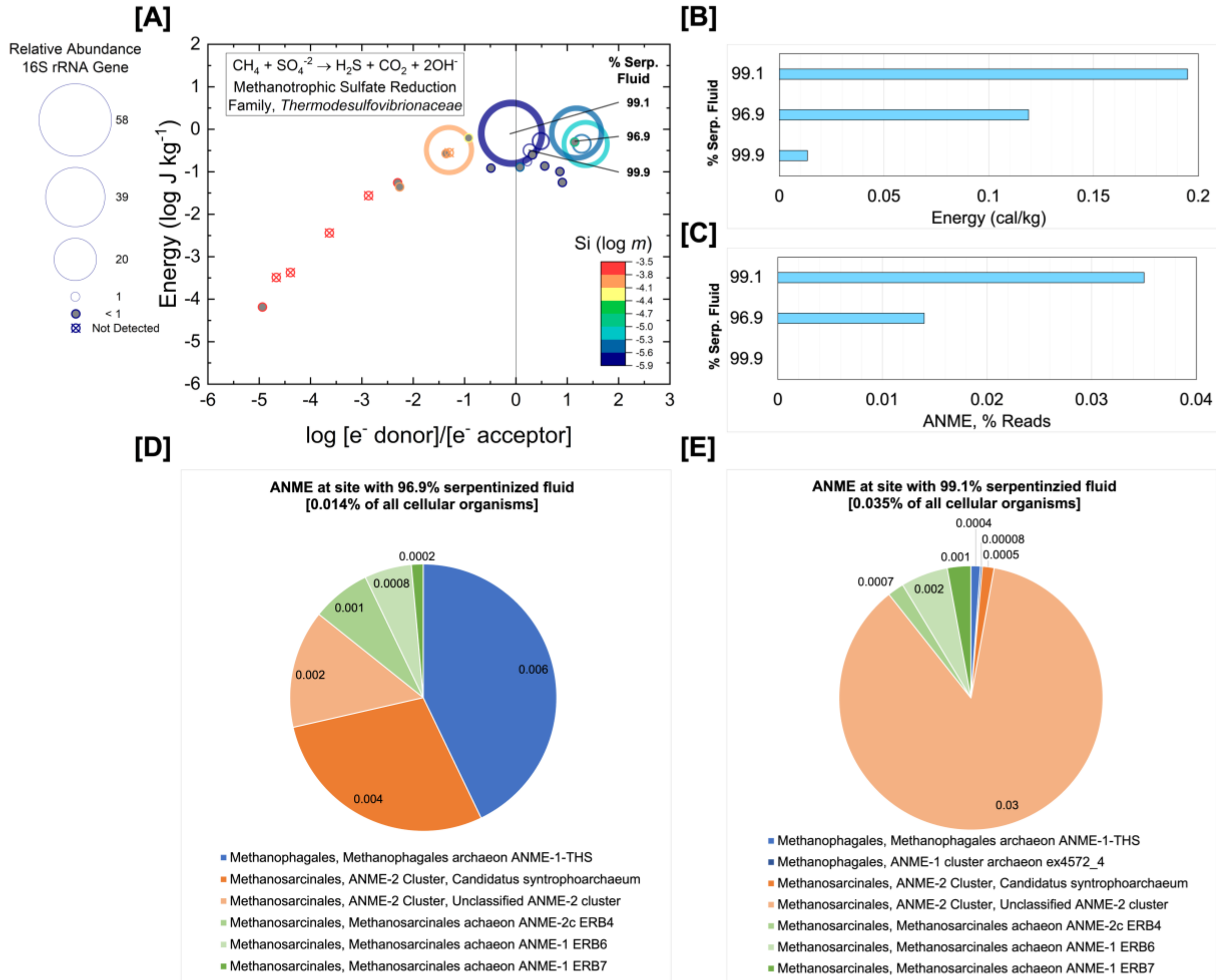

Supplementary Figure 3. Potential for anaerobic methane oxidation coupled to sulfate reduction. **[A]** The relative abundance of phylotypes classified as belonging to the family, *Thermodesulfovibrionaceae*, plotted as a function of the ratio of the electron donor (CH_4) to electron acceptor (SO_4^{-2}) corrected by the stoichiometry of the sulfate reduction reaction and the energy supply for sulfate reduction coupled to methane oxidation. *Thermodesulfovibrionaceae* are highlighted here as putative sulfate reducers and possible partners for anaerobic methane oxidation by ANME detected through annotations of raw shotgun metagenome sequencing reads. The three sampling sites where shotgun metagenome sequencing was conducted are highlighted by the % serpentinized fluid composition of the site fluid (99.1%, 140112K; 96.9%, 140111F; 99.9%, 140117H). **[B]** The energy supply for sulfate reduction coupled to methane oxidation of the three sampling sites where shotgun metagenome sequencing was conducted. **[C]** The % composition of the raw shotgun metagenome sequencing reads classified as ANME at the three shotgun metagenome sequencing sites. **[D]** The taxonomy of raw reads classified as ANME at site 140111F. **[E]** The taxonomy of raw reads classified as ANME at site 140112K.

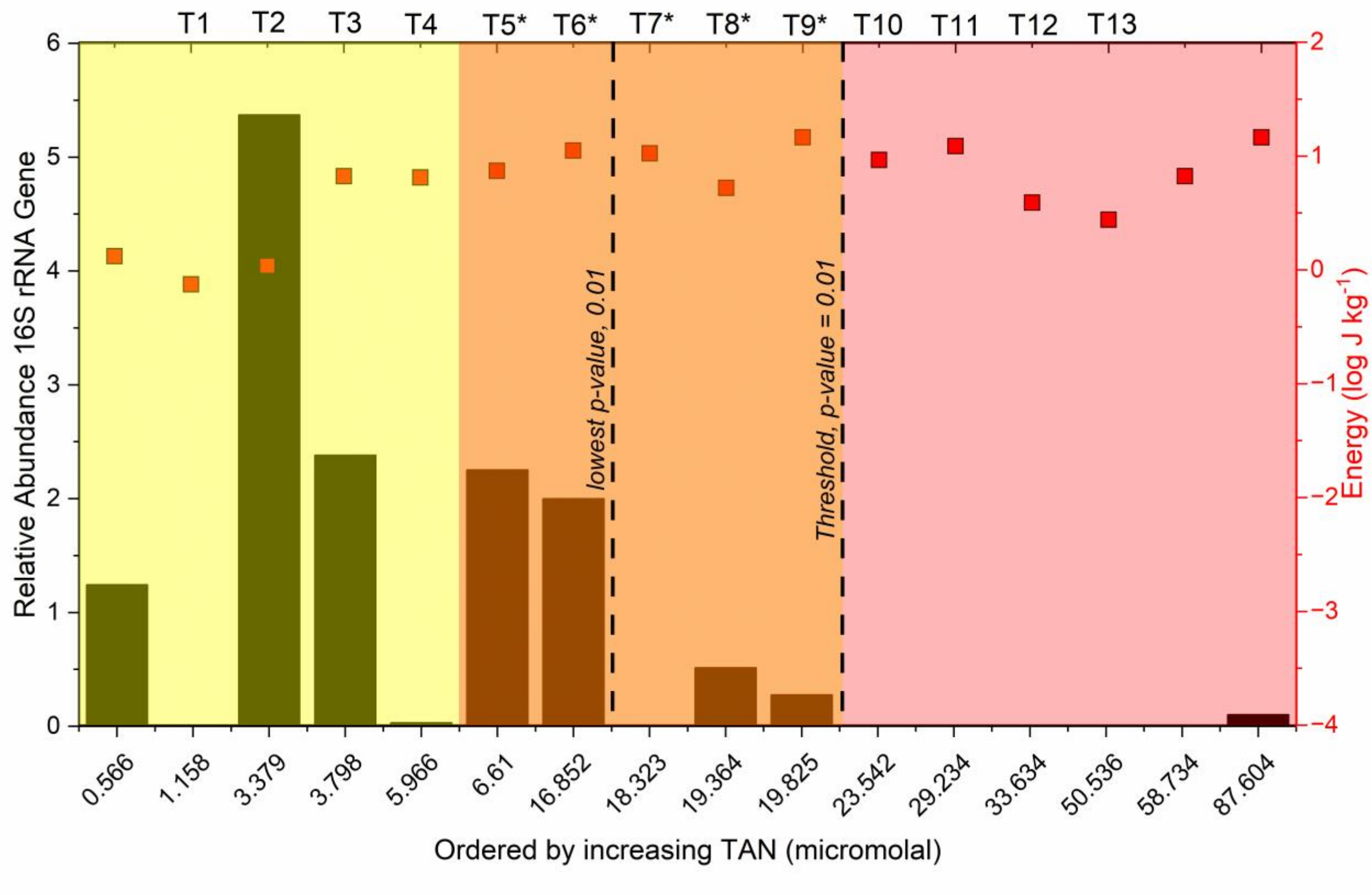

Supplementary Figure 4. Visual summary of Mann-Whitney U-tests to assess distribution of aerobic methanotrophs with respect to total ammonia nitrogen (TAN).

Exsolution Calculation.

To model the relative rates of $^{12}CH_4$ and $^{13}CH_4$ migration across the water-gas interface, we used the following equation,

$$\frac{dL}{dH} = \frac{1}{\alpha_k} \frac{(P\,\gamma - L_d)}{\left(P\,\gamma\,(\frac{H}{L})_g\,\alpha_{eq}\right) - \left(L_d\,(\frac{H}{L})_d\right)} \quad , \qquad (1)$$

where α_k is an experimentally determined kinetic isotopic fractionation factor, P is the average partial pressure of methane in the local atmospheric gas we measured, γ is the Henry's law constant for methane that we obtained from SUPCRT92 (Johnson et al., 1992), L_d is the concentration of the light isotope ($^{12}CH_4$) dissolved in solution, $(\frac{H}{L})_g$ is this average ratio of heavy to light methane in the local atmospheric gas, $(\frac{H}{L})_d$ is this ratio of heavy to light methane dissolved in solution, and α_{eq} is the equilibrium isotopic fractionation from dissolution. To constrain our upper bound for

isotopic fractionation during exsolution we used an α_k value of 0.9990, representing no turbulence, and to constrain our lower bound we used a value of 0.9994, representing a high level of turbulence (Knox et al., 1992). Our values for L_d and $(\frac{H}{L})_d$ for our upper bound were from sample 140115X while those values for our lower bound were from sample 140117F. Both sites that contained <1% shallow groundwater entrainment.

Rayleigh Calculation.

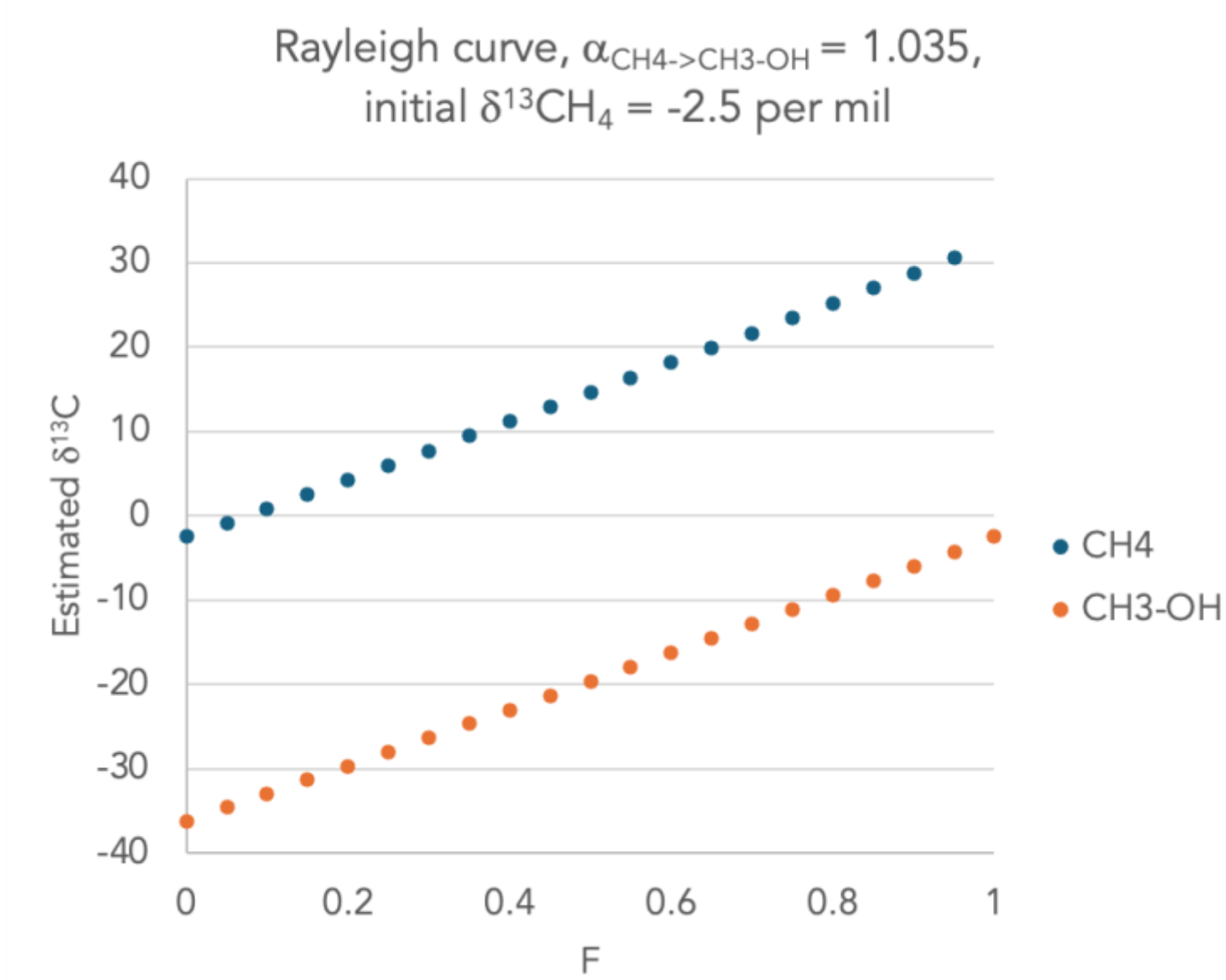

Supplementary Figure 5. Rayleigh calculations used for methanotrophy model in main text Figure 2b.

We implemented the Rayleigh equation (2) where δ_0 is the $\delta^{13}CH_4$ value of the source methane, which in this case is the highest value +1 SD of > 99% serpentinized fluids, -4.4 ± 1.9 ‰. The fractionation factor α (1.035) is for the conversion of CH_4 to CH_3OH in aerobic methanotrophs reported in Templeton et al. (2006). F is the fraction of methane pool consumed. We can therefore solve for δ_A, which is the estimated $\delta^{13}CH_3OH$ given the fraction of consumption. Using mass-balance (3) we can then solve for δ_B, which is the estimated $\delta^{13}CH_4$ value of the remaining pool.

$$\delta_A = (\delta_0 + 1000)/[F + \alpha(1-F)]-1000 \quad (2)$$

$$\delta_B = (F\delta_A - \delta_0)/(F - 1) \quad (3)$$
