# Supplementary material for: Methanotrophy under extreme alkalinity in a serpentinizing system
Source: Nat Commun. 2026 Jun 29;17:8135. doi: 10.1038/s41467-026-72513-6 (PMC13458360; doi:10.1038/s41467-026-72513-6)
Supplement: Supplementary file 3 [file 41467_2026_72513_MOESM2_ESM.pdf]

nature portfolio

Peer Review File

Methanotrophy Under Extreme Alkalinity in a Serpentinizing System

Corresponding Author: Dr Alta Howells

This file contains all reviewer reports in order by version, followed by all author rebuttals in order by version.

Version 0:

Reviewer comments:

Reviewer #1

(Remarks to the Author)
To the Authors:

I believe this manuscript is well-written and contributes a key finding to the fields of subsurface microbiology and astrobiology. There are some comments I have regarding clarification in certain areas of the manuscript, but overall the methodologies appear sound and I feel the conclusions are justified. I recommend this manuscript for publication with minor revision.

Summary:
This study aims to shed light on evidence for methanotrophy in the Samail Ophiolite in Oman, a serpentinizing system with hyperalkaline conditions. The evidence for methanotrophy in this environment is successfully presented via multiple methods, including genomics, isotope studies, and fluid mixing models. Although methanogenesis has been previously reported in these extreme environments previously, this manuscript presents a case for a contribution of methanotrophy as well. The presence and activity of this metabolic group has implications for our understanding of life in extreme environments, and should be considered when modeling other potential serpentinizing environments like those that may be on other Ocean Worlds.

Line notes:
Lines 46 – 47: I would consider a scarcity of carbon dioxide a potential nutrient limitation (depending on the total carbon of the system and which carbon sources are used for energy) rather than an extreme condition. Extreme conditions usually require specific genetic adaptations than enable cells to counteract the effects of the extreme, which I do not believe is the case here. I would consider rewording this to either reflect other extremes or that they are battling hyperalkaline conditions while also under nutrient limitation.

Line 61: The reference Leong et al., 2021 is in a different format than the rest.

Line 72: Can you briefly explain (1 – 2 sentences) how the changes in methane isotope values could be either explained by methane consumption or production? It’s unclear how these opposing processes can result in similar isotopic values (or are some positive, some negative?). This is a key part of interpreting the data presented in the manuscript and seems essential for interpreting results thus a bit of clarification would be helpful here.

Line 199: It’s good that this MAG contains expected pathways for methanotrophy; however, are there other pathways that can be added to this, such as the TCA cycle & glycolysis, which are also involved in carbon assimilation and biomolecule synthesis? Can you clearly state the carbon and energy sources for this predicted organism? Are they obtaining energy from methane oxidation and some carbon is assimilated into biomolecules, while other organic carbon sources are providing the rest? I would also like to a list of all annotated genes in addition to those only involved in these pathways. Could you please provide this in another supplementary table or the MAG assembly/scaffold as a fasta file – amino acid sequences or nucleotide – so that others could analyze the genome also?

Additionally, it does not seem this figure is presenting anything new or unexpected of methanotrophs that is directly supporting the claims in this manuscript. If this were to remain a figure in the main text instead of supplemental material,

consider either using it as a visual to help explain the flow of carbon and energy through the representative MAG in relation to the environment (see above for additional options) or state that this MAG has known pathways utilized by methanotrophs.

Line 201: Are enzyme names in black < 60% identity to methanotrophs or are they inferred? Please clarify.

Line 209: This MAG should be classified as a “high-quality draft” genome according to the standards set in this paper (Bowers et al., 2017): Bowers, R., Kyrpides, N., Stepanauskas, R. et al. Minimum information about a single amplified genome (MISAG) and a metagenome-assembled genome (MIMAG) of bacteria and archaea. Nat Biotechnol 35, 725–731 (2017). https://doi.org/10.1038/nbt.3893

Line 237: In the figure caption, d is used to denote delta instead of the symbol.

Line 263: Community composition or “methanotroph community structure” might be a more appropriate term to use here than alpha diversity. This figure does not present calculated values for alpha diversity or represent both species richness and evenness.

Line 272: Can you make some inferences as to why you are seeing variations in relative abundances of certain organisms along the gradient of serpentinized fluid? Is it relating to the energetics of the system and the presence of oxidants? Perhaps extending the final sentence to “…, which is consistent with our observation that …”?

Line 317: “…as reported in previous work.” should have a reference.

Reviewer #2

(Remarks to the Author)
This manuscript investigates the methane cycle in a serpentinized aquifer, with a particular focus on biological methane oxidation. The authors report several samples from the Oman ophiolite serpentinization field containing unusually $\delta^{13}CH_4$-enriched methane gas and explore possible mechanisms underlying this observation. In particular, biotic methane-oxidizing processes are examined as key factors shaping the methane cycle in serpentinized systems, underscoring their importance for understanding carbon cycling in these environments.

Major Comments

To prove the correlation of $\delta^{13}CH_4$-enriched methane gas with the microbial methane oxidation in the serpentinized system, more careful evidence is required.

1. Oxygen concentration is a critical factor controlling oxygen-dependent methane oxidation in serpentinitized waters. Since O_2 derived from Type I waters should be rapidly consumed by microbial or geochemical processes, its detection at well outlets is unexpected. But to strengthen your interpretation, please include field photographs or structural information of the wells. Such documentation would help readers assess whether the observed oxygen signals reflect in situ subsurface processes with shallow mixing or instead result from atmospheric contact. Since Si-based modeling alone cannot resolve this uncertainty, additional field documentation would be very valuable.

Separately, the labeling of the wells, particularly the use of alphabetic suffixes after the numbers (e.g., 140111G, H, I, and F), is unclear. Could you explain what these designations mean (e.g., different boreholes, casing types, or sampling depths)?

2. The whole story is based on only 140111 data. Don’t you have any other evidence to show the correlation of enrichment of both $\delta^{13}CH_4$-enriched and methanotrophs? Does this enrichment occur only in the specific well of 140111? If so, why? How about the other well? What are the geochemical or geological differences of 140111 and others?

3. It remains unclear where the methanotrophs are located within (inhabited) the system. Are they present in the water column, or in the sediment? Furthermore, what exactly is meant by “sediment” in this context? A serpentinization system is generally considered to consist of a water–rock interface, so the source of sediment is not immediately obvious. What materials are included in the sediment? Are there any differences between the microbial communities in the water and in the sediment? Could the sediment be a source of organic matter? Have you ever tried FISH to detect methanotroph?

4. (Line 179-180) MCR from ANME and pMMO are different enzymes. While you have cited the ANME example, it would also be important to address pMMO. Has $\delta^{13}CH_4$ enrichment been observed in this case, and is there supporting evidence? Furthermore, what is currently known about the remaining methane isotopologue signatures associated with pMMO? In addition, what is sodium concertation of this well?

5. Figure 2C. ANME and methanogens within the order Methanosarcinales share considerable genomic similarity. Because many core genes are conserved, relying solely on read mapping to MAGs or assemblies may blur the distinction between these groups. I recommend using SingleM to specifically identify the ANME fraction, as this tool uses single-copy marker genes for taxonomic assignment and can therefore provide a more robust separation of ANME from methanogenic Methanosarcinales. The same concern/consideration applies to Table 4.

6. Details on methanotrophs remain sparse. We recommend including (i) a phylogenetic tree to define their phylogenetic position and (ii) a map illustrating their global distribution in serpentinized systems.

7. Dima information is also limited. What are the geological (locational?) differences of 140112L , 140112M, 140110B and 140110D? Can you show the photos or geological information about the wells in the Dima?

8. Table 2; What is the phylotype of the 16S rRNA gene amplicon in the Table2? Broader context of aerobic methanotroph (like family level or genus level) or do you indicate specific phylotype of aerobic methanotroph? Please add 16S rRNA gene amplicon data of anaerobic archaean methanortroph to this table.

9. You may want to discuss for the other possible case to enrich $^{13}CH_4$ methane gas. In addition, if possible, you may want to theoritigally estimate how much portion of δ12CH4-methane must be consumed by the microbes to this extent of enrichment of δ13CH4-methane?

Minor comments

1. What is the O2 concentration of Type I endmember?
2. SO4-2 to SO42-

Open Access This Peer Review File is licensed under a Creative Commons Attribution 4.0 International License, which permits use, sharing, adaptation, distribution and reproduction in any medium or format, as long as you give appropriate credit to the original author(s) and the source, provide a link to the Creative Commons license, and indicate if changes were made.
In cases where reviewers are anonymous, credit should be given to 'Anonymous Referee' and the source.
The images or other third party material in this Peer Review File are included in the article's Creative Commons license, unless indicated otherwise in a credit line to the material. If material is not included in the article's Creative Commons license and your intended use is not permitted by statutory regulation or exceeds the permitted use, you will need to obtain permission directly from the copyright holder.
To view a copy of this license, visit https://creativecommons.org/licenses/by/4.0/

Dear Reviewers,

Responses to your comments are in blue. Thank you for your feedback. I have done by best to address your suggestions.

Kind Regards,
Corresponding Author

REVIEWER COMMENTS

Reviewer #1 (Remarks to the Author):

To the Authors:

I believe this manuscript is well-written and contributes a key finding to the fields of subsurface microbiology and astrobiology. There are some comments I have regarding clarification in certain areas of the manuscript, but overall the methodologies appear sound and I feel the conclusions are justified. I recommend this manuscript for publication with minor revision.

Summary:
This study aims to shed light on evidence for methanotrophy in the Samail Ophiolite in Oman, a serpentinizing system with hyperalkaline conditions. The evidence for methanotrophy in this environment is successfully presented via multiple methods, including genomics, isotope studies, and fluid mixing models. Although methanogenesis has been previously reported in these extreme environments previously, this manuscript presents a case for a contribution of methanotrophy as well. The presence and activity of this metabolic group has implications for our understanding of life in extreme environments, and should be considered when modeling other potential serpentinizing environments like those that may be on other Ocean Worlds.

Line notes:
Lines 46 – 47: I would consider a scarcity of carbon dioxide a potential nutrient limitation (depending on the total carbon of the system and which carbon sources are used for energy) rather than an extreme condition. Extreme conditions usually require specific genetic adaptations than enable cells to counteract the effects of the extreme, which I do not believe is the case here. I would consider rewording this to either reflect other extremes or that they are battling hyperalkaline conditions while also under nutrient limitation.
Thank you for this suggestion. I have addressed this by removing the word ‘polyextreme’ at line 47 and removed ‘poly’ from ‘extreme’ in line 33.

Line 61: The reference Leong et al., 2021 is in a different format than the rest.
I have changed this to reference 3: Templeton, A. S. et al. Low-temperature hydrogen production and consumption in partially-hydrated peridotites in Oman: implications for stimulated geological hydrogen production. Front. Geochem. 2, 1366268 (2024).

Line 72: Can you briefly explain (1 – 2 sentences) how the changes in methane isotope values could be either explained by methane consumption or production? It's unclear how these opposing processes can result in similar isotopic values (or are some positive, some negative?). This is a key part of interpreting the data presented in the manuscript and seems essential for interpreting results thus a bit of clarification would be helpful here.
I have added details on how this can happen and added a reference (line 82-84): Batther, H. K., Templeton, A. S., Hoehler, T., Howells, A., Bill, M., Gropp, J., et al. (2025). The stable carbon isotope fractionation of methanogenesis products at complete carbon consumption. Geochem. Persp. Let. 37, 35–39. doi: 10.7185/geochemlet.2543

Line 199: It's good that this MAG contains expected pathways for methanotrophy; however, are there other pathways that can be added to this, such as the TCA cycle & glycolysis, which are also involved in carbon assimilation and biomolecule synthesis? Can you clearly state the carbon and energy sources for this predicted organism? Are they obtaining energy from methane oxidation and some carbon is assimilated into biomolecules, while other organic carbon sources are providing the rest? I would also like to a list of all annotated genes in addition to those only involved in these pathways. Could you please provide this in another supplementary table or the MAG assembly/scaffold as a fasta file – amino acid sequences or nucleotide – so that others could analyze the genome also?
I conducted a DRAM analysis with results added to a new supplementary file (Supp. Table 7, amino acid sequences and their annotations in Supplementary File 2) which shows evidence for both glycolysis and the TCA cycle. In other methanotrophs D-fructose-1,6-P2 generated in the RuMP Cycle (formaldehyde fed in from methane oxidation) can feed into glycolysis to generate pyruvate producing ATP and NADP. Pyruvate can then feed into the TCA cycle for generation of NADH and GTP. I've included this proposed energy generation mechanism into Figure 3. Please let me know if this is clear enough. I would like to note that these are proposed carbon assimilation and energy generation pathways based on gene presence. A deeper analysis of the genetic repertoire, and potentially metabolic flux modeling, could be done, however I believe this could go in a separate paper given the number of figures and length of text of this paper. For readers interested in a deeper analysis I have noted that I will send those interested in the .fasta file of the MAGs immediately upon request. The raw sequencing reads are available through NCBI SRA as noted in the data availability statement. I may be able to upload the MAGs as genomes to SRA if it meets their cutoff for genome quality. Please let me know if you would like me to do so.

Additionally, it does not seem this figure is presenting anything new or unexpected of methanotrophs that is directly supporting the claims in this manuscript. If this were to remain a figure in the main text instead of supplemental material, consider either using it as a visual to help explain the flow of carbon and energy through the representative MAG in relation to the environment (see above for additional options) or state that this MAG has known pathways utilized by methanotrophs.
Figure 3 now emphasizes three points:

1) Fractionation of methane occurs upon the uptake of methane into cell, and subsequent oxidation of methane to methanol
2) Methanol is reduced to formaldehyde which can then feed into pathways for carbon assimilation and energy generation from methane alone
3) Presence of Na+/H+ antiporters that in other organisms are known to be adaptations to alkaline conditions.

Therefore, I would like to keep the figure in the main text.

Line 201: Are enzyme names in black < 60% identity to methanotrophs or are they inferred? Please clarify.
I have noted in Figure 3 caption: Those in black were not detected in the MAGs through our annotation method.

Line 209: This MAG should be classified as a "high-quality draft" genome according to the standards set in this paper (Bowers et al., 2017): Bowers, R., Kyrpides, N., Stepanauskas, R. et al. Minimum information about a single amplified genome (MISAG) and a metagenome-assembled genome (MIMAG) of bacteria and archaea. Nat Biotechnol 35, 725–731 (2017). https://doi.org/10.1038/nbt.3893
At line 228 I have added a note that Bin39 is a high-quality draft genome.

Line 237: In the figure caption, d is used to denote delta instead of the symbol.
Thank you for bringing this to my attention. I have corrected this error.

Line 263: Community composition or "methanotroph community structure" might be a more appropriate term to use here than alpha diversity. This figure does not present calculated values for alpha diversity or represent both species richness and evenness.
I have changed the text to say methanotroph community structure. Thank you for the recommendation.

Line 272: Can you make some inferences as to why you are seeing variations in relative abundances of certain organisms along the gradient of serpentinized fluid? Is it relating to the energetics of the system and the presence of oxidants? Perhaps extending the final sentence to "…, which is consistent with our observation that …"?
The section following this line goes into detail on what we hypothesize influences their relative abundance. Figure 6 summarizes our observations.

Line 317: "…as reported in previous work." should have a reference.
I have added references. Thank you for bringing this to my attention.

Reviewer #2 (Remarks to the Author):

This manuscript investigates the methane cycle in a serpentinized aquifer, with a particular focus on biological methane oxidation. The authors report several samples from the Oman ophiolite serpentinization field containing unusually $\delta^{13}CH_4$-enriched methane gas and explore possible mechanisms underlying this observation. In

particular, biotic methane-oxidizing processes are examined as key factors shaping the methane cycle in serpentinized systems, underscoring their importance for understanding carbon cycling in these environments.

Major Comments

To prove the correlation of $\delta^{13}CH_4$-enriched methane gas with the microbial methane oxidation in the serpentinized system, more careful evidence is required.

1. Oxygen concentration is a critical factor controlling oxygen-dependent methane oxidation in serpentinitized waters. Since O_2 derived from Type I waters should be rapidly consumed by microbial or geochemical processes, its detection at well outlets is unexpected. But to strengthen your interpretation, please include field photographs or structural information of the wells. Such documentation would help readers assess whether the observed oxygen signals reflect in situ subsurface processes with shallow mixing or instead result from atmospheric contact. Since Si-based modeling alone cannot resolve this uncertainty, additional field documentation would be very valuable.
The field sites are surface expressions as mentioned at line 412. Therefore, there is a supply of O2 from the atmosphere. O2 is lower at the surface expression source and increases with mixing and down outflows as shown in previous publications (Howells et al., 2022; Howells et al., 2023). Please see Supplementary Table 1 for all O2 concentrations as well as other relevant chemical constituents.

While readers can read Howells et al. (2022) for further details on fluids at the surface and how geochemistry changes along the mixing gradient, I have included a detailed overview of the geochemistry, methane isotopes, field images and biology of the Qafifah location Supplementary Figure 1. This also addresses comment 2.

Separately, the labeling of the wells, particularly the use of alphabetic suffixes after the numbers (e.g., 140111G, H, I, and F), is unclear. Could you explain what these designations mean (e.g., different boreholes, casing types, or sampling depths)?
The different date codes represent different sampling sites.

2. The whole story is based on only 140111 data. Don't you have any other evidence to show the correlation of enrichment of both $\delta^{13}CH_4$-enriched and methanotrophs? Does this enrichment occur only in the specific well of 140111? If so, why? How about the other well? What are the geochemical or geological differences of 140111 and others?
To make the connection between d13CH4 enrichment and methanotrophy more clear I have include supplementary figure…
Please see response to comment 1. Evidence of methanotrophy corresponding to delta13CH4 enrichment is also shown in Figure 2 and Figure 5 (16S rRNA gene amplicons). I request that you take a moment to review Figure 2 and Table 2.

3. It remains unclear where the methanotrophs are located within (inhabited) the system. Are they present in the water column, or in the sediment? Furthermore, what exactly is meant by "sediment" in this context? A serpentinization system is generally

considered to consist of a water–rock interface, so the source of sediment is not immediately obvious. What materials are included in the sediment? Are there any differences between the microbial communities in the water and in the sediment? Could the sediment be a source of organic matter? Have you ever tried FISH to detect methanotroph?

I have added a description of the sediment in the methods section at lines 425-426.

4. (Line 179-180) MCR from ANME and pMMO are different enzymes. While you have cited the ANME example, it would also be important to address pMMO. Has $\delta^{13}CH_4$ enrichment been observed in this case, and is there supporting evidence? Furthermore, what is currently known about the remaining methane isotopologue signatures associated with pMMO? In addition, what is sodium concertation of this well?

We stated at line 264 we were not able to resolve a MAG for ANME. However, upon conducting taxonomic assignment of raw sequencing reads using the program Kaiju (uses HMMs like SingleM) we find some evidence for ANME1 and ANME2, although sparse. To emphasize the low confidence in this finding I have added a "?" to ANME in Figure 4c. Furthermore, ANME 16S rRNA gene sequences were not detected through our 16S rRNA gene amplicon sequencing and analysis approach as stated at line 498. While these results are not definitive, we would still like to include them to highlight the potential for anaerobic methane oxidation in the subsurface where there might be more methane available, but we are currently not able to sample due to the lack of wells in this region. The potential for anaerobic methane oxidation is further examined in Supplementary Figure 3.

For aerobic methanotrophs we have evidence of their presence through the 16S rRNA gene amplicons and through shotgun metagenome sequencing. I noted the presence of pMMO genes in the aerobic methanotroph MAGs (classified as *Methylovolum*) at line 234. Genes and their annotations can be found in Supplementary Table 6 and Supplementary File 2.

I unfortunately do not understand this question as this paper is focused on 13CH4 and 12CH4: "Furthermore, what is currently known about the remaining methane isotopologue signatures associated with pMMO?"

5. Figure 2C. ANME and methanogens within the order Methanosarcinales share considerable genomic similarity. Because many core genes are conserved, relying solely on read mapping to MAGs or assemblies may blur the distinction between these groups. I recommend using SingleM to specifically identify the ANME fraction, as this tool uses single-copy marker genes for taxonomic assignment and can therefore provide a more robust separation of ANME from methanogenic Methanosarcinales. The same concern/consideration applies to Table 4.

https://www.nature.com/articles/s41587-025-02738-1

Thank you for this recommendation. As I state in the response to comment 4, the potential presence of ANME is based on annotation of the raw sequencing reads using

the program Kaiju, which like SingleM uses HMMs of amino acid sequences. We used this approach given that we were not able to resolve a MAG for ANME, suggesting that if ANME are there, they are in very low abundance at the sampling sites of Dima. We also note in the text that the enriched 13CH4 observed in Dima fluids may also be the result of anaerobic methanotrophy in the subsurface before the fluids are expressed at the surface, something we could not examine in this study given our focus on surface expressed fluids. Previous studies on Oman well and borehole fluids do not have such CH4-rich fluids as Dima and therefore, this metabolism may be missed in those field sites. Future exploration of the subsurface in Oman could target regions for drilling were CH4-rich surface expressed fluids like those of Dima are located.

6. Details on methanotrophs remain sparse. We recommend including (i) a phylogenetic tree to define their phylogenetic position and (ii) a map illustrating their global distribution in serpentinized systems.
This study is focused on methanotrophy in serpentinized fluids of Oman. While I think this is an excellent recommendation, given the synthesis of isotopic, geochemical and biological datasets, the number of figures and length of text of the current version of the manuscript, I believe such an analysis could go in a separate paper and would provide meaningful insights into hyperalkaliphilic methanotrophy across the globe. Some of the confusion may be from the title of this manuscript. I can change it to be more specific to Oman, "Methanotrophy Under Extreme Alkalinity in Surface Expressions of Serpentinized Fluids in the Samail Ophiolite".

7. Dima information is also limited. What are the geological (locational?) differences of 140112L , 140112M, 140110B and 140110D? Can you show the photos or geological information about the wells in the Dima?
Please see response to comment 1.

8. Table 2; What is the phylotype of the 16S rRNA gene amplicon in the Table2? Broader context of aerobic methanotroph (like family level or genus level) or do you indicate specific phylotype of aerobic methanotroph? Please add 16S rRNA gene amplicon data of anaerobic archaean methanortroph to this table.
Please see response to comment 4 regarding ANME amplicons. For further details on the aerobic methanotroph phylotypes, see Supplementary Tables 4, 5 and 6.

9. You may want to discuss for the other possible case to enrich $^{13}CH_4$ methane gas. In addition, if possible, you may want to theoritigally estimate how much portion of δ12CH4-methane must be consumed by the microbes to this extent of enrichment of δ13CH4-methane?
Although we had already addressed this with an enrichment arrow in Figure 2B, I have added methanotrophy contours to Figure 2B to make it more clear.

Minor comments

1. What is the O2 concentration of Type I endmember?
2. SO4-2 to SO42-

Please see Supplementary Table 1 for O2 and SO4-2 concentrations.
