# Supplementary material for: Methanotrophy under extreme alkalinity in a serpentinizing system
Source: Nat Commun. 2026 Jun 29;17:8135. doi: 10.1038/s41467-026-72513-6 (PMC13458360; doi:10.1038/s41467-026-72513-6)
Supplement: Supplementary file 4 [file 41467_2026_72513_MOESM3_ESM.pdf]

Description of Additional Supplementary Files

Supplementary Data 1. Concentrations of reactants and products for methane oxidation coupled to O2, NO3- and SO3-2. Estimated energy availability in unite kJ per mole electron and kJ per kg fluid.

Supplementary Data 2. Core methanotroph metabolism genes, annotations and BLASTp results.

Supplementary Data 3. Descriptive statistics and Mann-Whitney U test parameters to assess the significance of distribution differences of aerobic methanotroph phylotypes with respect to total ammonia nitrogen (TAN).

Supplementary Data 4. ASVs classified as methanotrophs, the taxonomic assignment and BLASTn results.

Supplementary Data 5. Relative abundance of ASVs classified as methanotrophs.

Supplementary Data 6. Relative abundance of methanotroph phylotypes by group.

Supplementary Data 7. Bin39 metabolic pathway overview from DRAM annotation.

Supplementary Code 1. .faa file for coding sequences of Bin39 and their DRAM annotations.
